# From Water Delivery to Root-Zone Engineering: Drip Irrigation Drives Adaptive Root Architectural Plasticity and Enhances Spatiotemporal Root–Water Coupling in Maize

**DOI:** 10.3390/plants15131978

**Published:** 2026-06-26

**Authors:** Bin Wang, Licun Zhang, Guodong Wang, Jiliang Zheng, Fei Liang

**Affiliations:** 1 School of Resources and Environment, Yili Normal University, Yining 835000, China; 2 Institute of Resources and Ecology, Yili Normal University, Yining 835000, China; 3 Institute of Farmland Water Conservancy and Soil-Fertilizer, Xinjiang Academy of Agricultural Reclamation Science, Shihezi 832000, China; 4 Xinjiang Xinlianxin Energy Chemical Co., Ltd., Changji 832200, China; 5 Yili River Valley Agricultural Resources and Environment Laboratory, Yili Normal University, Yining 835000, China

**Keywords:** root-water spatial coupling, root-zone engineering, fine-root proliferation, drought-root plasticity nexus, water productivity in arid agriculture

## Abstract

The shift from furrow irrigation (FI) to mulched drip irrigation (DI) drives adaptive restructuring of maize root architecture, strengthening spatiotemporal root-soil water coupling. Conventional FI often leads to a spatial mismatch between root distribution and water supply, constraining water productivity in arid regions. Using a three-year field experiment (2019–2021) in Xinjiang, China, this study examined how DI reshapes maize root architecture in three dimensions and affects grain yield under contrasting hydrological conditions. Results demonstrate that DI systematically reconfigures root spatial deployment, promoting a “fine-and-dense” root phenotype. Compared to FI, DI increased fine and medium root length by 35.0–38.6% in the emitter-proximal zone (0–10 cm) and concentrated 78.2% of absorptive roots within the upper 0–20 cm. Vertical root distribution followed an exponential decay pattern, with significantly steeper gradients under DI-root length at 10 cm depth was 303.4% (2019) and 50.4% (2020) higher than under FI (*p* < 0.05). In contrast, FI promoted deeper root exploration, showing a 49.1–66.1% advantage at 40 cm depth. Root restructuring proved hydrologically conditional: DI increased root length density (RLD) by 224.6% under drought conditions (2019) but only by 29.1% under ample moisture (2021). This trend reversed at the R3 stage, where FI achieved 117.2% higher RLD than DI (*p* < 0.05). Importantly, topsoil RLD mediated the pathway from irrigation to yield (R^2^ = 0.87, *p* < 0.001), identifying spatial root–water coupling as the dominant mechanism. These findings advance irrigation management by accounting for root architectural responses to localized water delivery, providing theoretical guidance for precision agriculture in arid environments.

## 1. Introduction

Maize (*Zea mays* L.) serves as a staple crop in arid regions worldwide, yet faces escalating drought stress due to climate change and limited freshwater resources [1,2]. In Northwest China, particularly Xinjiang, the multi-year average precipitation is only 157.7 mm, while potential evapotranspiration exceeds 1000 mm annually [3]. These abiotic constraints result in significant yield variability, threatening regional food security [4,5]. Irrigation remains the primary mitigation strategy; however, conventional furrow irrigation exhibits low water use efficiency, with water productivity averaging only 1.2–1.5 kg m^−3^ in arid regions [6]. Consequently, developing innovative technologies that enhance soil water retention and achieve spatiotemporal coupling between root systems and water availability is critical for sustainable maize production [4,7,8].

Root systems function as the “hidden half” of plants, providing anchorage and regulating water and nutrient uptake, and serving as the primary interface with environmental stimuli [9,10]. Root morphology and spatial distribution directly influence crop resource acquisition, with architectural plasticity representing a key adaptive mechanism for drought resistance [11,12,13]. Specifically, Yu et al. [11] demonstrated that maize lateral root branching plasticity enables selective proliferation in nutrient-rich patches, increasing root length density by 15–30% under heterogeneous conditions. Liu et al. [12] further reported that plastic redistribution of roots in the 0–20 cm layer contributes to 20–35% recovery in maize growth during late developmental stages under intercropping systems. Under water deficit, maize roots optimize morphological traits to enhance water extraction from deeper soil profiles [14,15], whereas adequate moisture promotes vigorous shallow proliferation [16,17]. Recent advances in root phenotyping reveal that genotype × environment interactions strongly modulate root plasticity, with modern hybrids showing greater responsiveness to precision irrigation than traditional varieties [18,19]. Root architectural plasticity is mediated by active hormone signaling rather than passive physical responses. Hydrotropism directs root growth toward water via MIZ1-dependent Ca^2+^ signaling, while hydropatterning preferentially positions lateral roots toward moist soil through PIN3-auxin transport. These mechanisms enable roots to actively forage for water rather than merely grow where water is physically available [20,21,22].

Water scarcity critically limits agricultural sustainability in Northwest China [23]. Drip irrigation under plastic film mulch (DI) synergistically combines precise water delivery with evaporation suppression, creating optimized root-zone microenvironments that enhance water-heat-nutrient interactions [24,25]. By supplying water directly to the root-dense layer (0–20 cm) via emitters, DI reduces soil evaporation by 30–50% and deep percolation by 20–40% compared to flood irrigation [6,26], Qu et al. [26] further reported that DI coupled with controlled-release potassium fertilizer increased maize yield by 12.5% and water use efficiency by 18.3% in a two-year field trial. DI increases water productivity by 18.5–44.0% in crops such as cotton, grape, and tomato [7,25], yet its impact on maize root spatial architecture remains insufficiently quantified [4,27,28].

Emerging research demonstrates that DI enhances root proliferation in shallow soil horizons (0–20 cm), while exhibiting progressive attenuation at greater depths, but empirical evidence linking soil moisture heterogeneity, vertical root plasticity, and maize water use efficiency remains scarce [23]. Emerging research emphasizes that RLD and RSAD are key determinants of water and nutrient uptake and yield formation [10,27]. Furthermore, the spatiotemporal coupling between root distribution and soil water availability determines crop performance under deficit irrigation [5,8,29]. The integration of smart irrigation systems with real-time root monitoring offers new opportunities for optimizing water productivity. However, the mechanistic basis of how drip irrigation-driven soil moisture dynamics reshape maize root system architecture—and consequently yield—requires systematic investigation across hydrologically contrasting scenarios [4,30].

This investigation seeks to address the following questions: (1) how the transition from furrow to drip irrigation reshapes root diameter-class distribution and spatial deployment; (2) the hydrological context-dependency of root plasticity responses; and (3) what mechanistic pathways link irrigation-induced root restructuring to yield formation. By integrating root phenotyping with yield performance over three years (2019–2021), we provide evidence-based guidance for precision water management in arid agriculture. We hypothesize that (1) drip irrigation induces a shallow-concentrated root phenotype through hydrotropism and hydropatterning responses to localized wetting fronts; (2) this architectural plasticity reduces the metabolic cost of soil exploration by concentrating absorptive roots within the wetted volume; and (3) enhanced root-water spatial coupling mediates yield improvement under water deficit. To test whether irrigation-driven soil moisture heterogeneity actively reshapes root architecture through hormonal signaling pathways, a three-year field experiment was conducted to quantify root architectural responses and their association with yield formation.

## 2. Materials and Methods

### 2.1. Experimental Sites and Soil Conditions

**Figure 1:**
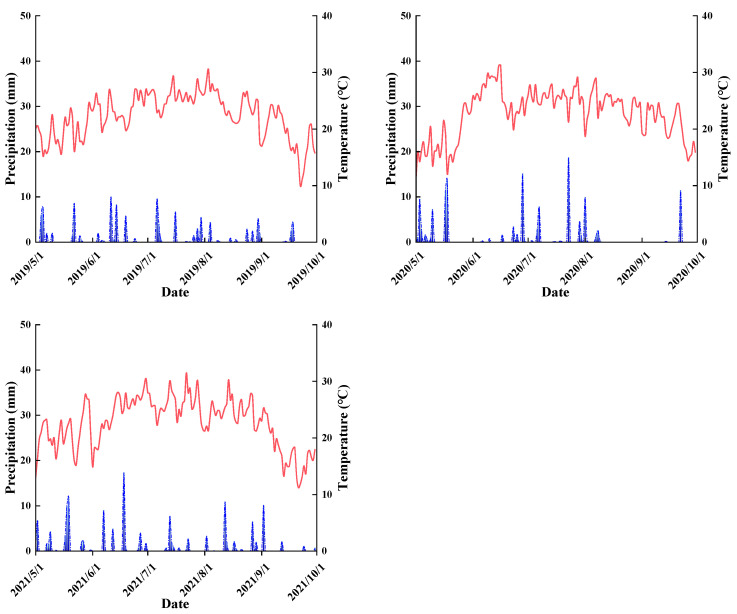
Daily precipitation and mean air temperature during the 2019–2021 growing seasons (Red lines temperature, and blue bars represent precipitation).

This study was conducted from 2019 to 2021 at the Scientific Observation and Experiment Station of Crop High Efficiency Water Use, Ministry of Agriculture and Rural Affairs, located in Shihezi City, Xinjiang (45°38′ N, 86°09′ E). The experimental site is situated on an alluvial fan plain at the northern foothills of the Tianshan Mountains. The tested soil is gray desert soil. The basic physicochemical properties of the soil are shown in Table 1. The site is characterized by a typical temperate continental climate. During the study period, maximum and minimum air temperatures were 30.9 °C and 14.9 °C, respectively. The mean temperature during the maize growing season (May–September) was 22.6 °C, with average precipitation of 130.6 mm (Figure 1). The groundwater depth ranged from 1.2 to 1.8 m.

### 2.2. Experimental Design

**Figure 2:**
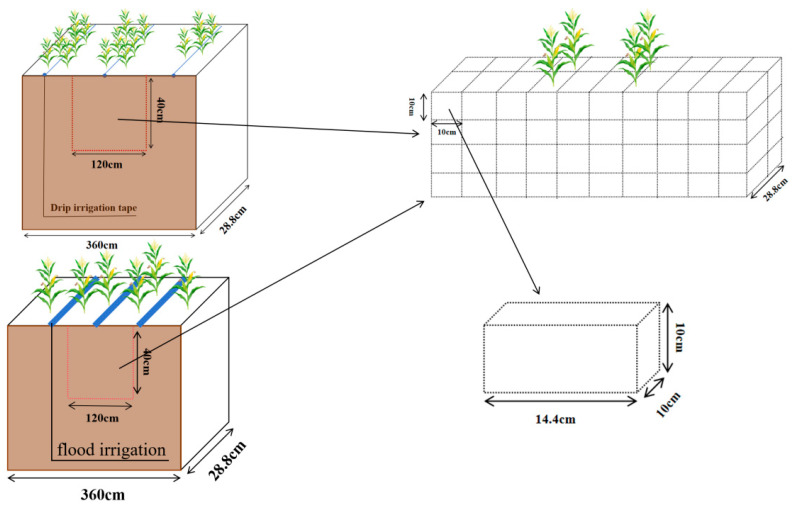
Schematic diagram of the maize root sample (10 cm × 10 cm × 14.4 cm) collected between 2019 and 2021.

This experiment compared two treatments: DI and FI. Both DI and FI received an identical irrigation amount of 4800 m^3^ ha^−1^. The experimental design was a randomized complete block with three replications, and each plot had an area of 200 m^2^. Maize was sown in an alternating narrow-wide row spacing of 30 and 80 cm, following a planting mode of one plastic mulch sheet covering two crop rows served by one drip line. Plant spacing within rows was 14.4 cm, achieving a planting density of 115,500 plants ha^−1^. Each treatment was equipped with an independent fertigation system, and irrigation water volumes were measured precisely using water meters. Fertilizer application rates were as follows: urea (N ≥ 46.4%, granular; Xinjiang Xinlianxin Co., Ltd., Xinjiang, China) at 720 kg ha^−1^, monoammonium phosphate (N ≥ 12%, P_2_O_5_ ≥ 61%, powder; Guizhou Kai Phosphorus Group Co., Ltd., Guiyang, China) at 255 kg ha^−1^, and potassium sulphate (K2O ≥51%, granular; Xinjiang Lop Nur Potassium Salt Co., Ltd., Xinjiang, China) at 330 kg ha^−1^. The field trial used the maize cultivar ‘Zhengdan 958’, which provided by Beijing Denong Seed Technology Co., Ltd. (Beijing, China). Built-in drip tape was employed (inner diameter: 16 mm; wall thickness: 0.18 mm; Xinjiang Drip Irrigation, Sprinkler Irrigation and Water Pipe Water-saving Equipment Co., Ltd., Xinjiang, China), with emitters spaced at 300 mm and a flow rate of 2.0 L h^−1^ per emitter under an operating pressure of 0.15 MPa. Sowing occurred on 2 May 2019, 30 April 2020, and 7 May 2021. A schematic diagram of the maize cultivation pattern is presented in Figure 2.

### 2.3. Root Sampling and Measurements

The maize root system was defined as the portion below the soil surface. Root samples were collected using a systematic spatial grid sampling based on a systematic spatial grid. Within the area occupied by four maize plants (120 cm along the row × 28.8 cm perpendicular to rows, corresponding to alternating 30 and 80 cm row spacing), soil was vertically sliced in 10 cm increments from the surface to 40 cm depth (Figure 2). A custom-made root sampler was used to extract rectangular soil blocks measuring 14.4 cm (along row) × 10 cm (depth) × 10 cm (across row). The four plants were each positioned at the center of one sampling unit, yielding 20 blocks per quadrat across four depth layers (0–10, 10–20, 20–30, and 30–40 cm) and five lateral positions (0–10, 10–20, 20–30, 30–40 and 40–50 cm from the plant row), totaling 60 blocks per sampling date (20 blocks × 3 replications), or 120 blocks across both sampling dates (V6 and R3) Root sampling was limited to 0–40 cm because preliminary surveys showed >90% of maize root length was distributed above 40 cm, where penetration resistance of the compacted subsoil (>2.5 MPa) restricted further elongation.

Sampling was conducted at the jointing (V6) and milk (R3) growth stages. For each treatment, two sets of root-soil blocks were collected: one set was manually washed to separate roots for physiological analysis; the other was scanned using the WinRHIZO root analysis system (Regent Instruments Inc., Quebec, QC, Canada) to determine root length, surface area, volume, and diameter class distribution. Following imaging, roots were oven-dried at 75 °C to constant weight (approximately 48 h) to determine root dry mass.

RLD refers to the total root length per unit volume of soil:

RLD = L/V

where RLD is the root length density (cm/cm^3^), L is the total root length (cm), and V is the soil volume (cm^3^).

Root surface area density (RSAD) refers to the total root surface area per unit volume of soil:

RSAD = RSA/V

where RSAD is the root surface area density (cm^2^/cm^3^), RSA is the total root surface area (cm^2^), and V is the soil volume (cm^3^).

Root volume density (RVD) refers to the total root volume per unit volume of soil:

RVD = RV/V

where RVD is the root volume density (cm^3^/cm^3^), RV is the total root volume (cm^3^), and V is the soil volume (cm^3^).

The root-to-shoot ratio (R/S) is calculated as the ratio of belowground root dry mass to aboveground shoot dry mass.

### 2.4. Yield and Yield Composition

Twenty ears were randomly selected from each plot. Their fresh weights were measured, followed by air-drying and seed quality inspection. The grain yield per unit area was calculated at a standard moisture content of 14%. The number of effective plants, ears per plant, grains per row, rows per ear, and thousand-grain weight were investigated to analyze the yield components [31]:

Yield (kg·hm^−2^) = [Grain weight of 20 ears (g)/Number of ears of 20 plants] × (115,500/1000) × [(1 − Grain moisture content (%))/(1 − 14%)]

The standard moisture content is 14%, and the planting density is 115,500 plants per hectare.

### 2.5. Data Analysis

All data were analyzed using IBM SPSS Statistics 26 and Excel 2019. Graphs were generated with Origin 2024. Significant differences among treatments were determined by least significant difference (LSD) multiple range tests.

Root-water spatial coupling was quantified using the Sørensen similarity index (SSI) to measure spatial congruence between RLD and soil water content (SWC) distributions across the 20 grid cells (4 depths × 5 lateral positions) under mulched DI and FI:

SSI = [2 × Σ(min(RWi, SWi))]/(ΣRWi + ΣSWi)

where RWi = RLDi/ΣRLDi and SWi = SWCi/ΣSWCi for the i-th grid cell. SSI ranges from 0 (complete spatial mismatch) to 1 (perfect overlap).

## 3. Results

### 3.1. Irrigation Regime Alters Soil Water Distribution

**Figure 3:**
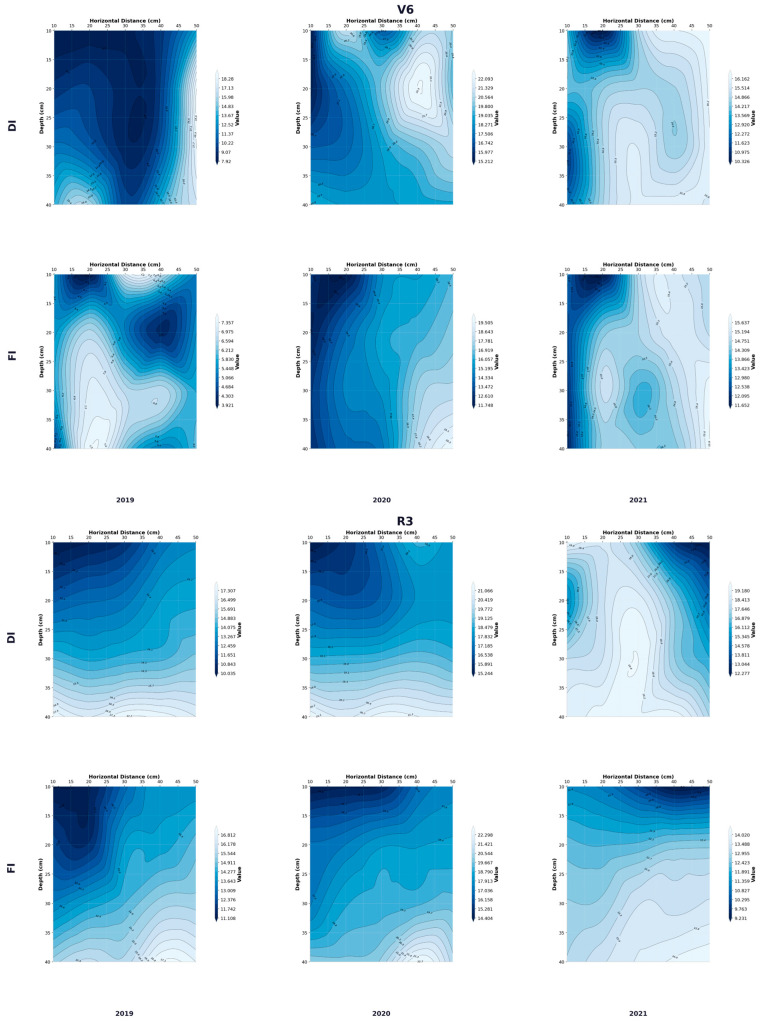
Spatial Distribution of Soil Water Content during V6 and R3 Growth Stages.

The spatiotemporal distribution of soil water content under contrasting irrigation regimes exhibited distinct hydrological signatures across the three experimental years (Figure 3). At the V6 stage, DI generated pronounced soil moisture heterogeneity, with water content peaking in the emitter-proximal zone (0–20 cm horizontal distance, 0–20 cm depth) and declining exponentially with both horizontal and vertical distance from the drip line. This localized wetting pattern was most pronounced in the drought year of 2019, where DI maintained 18.3% soil water content in the near-emitter zone compared to 7.9% in the distant zone (40–50 cm), creating a steep moisture gradient of 10.36 percentage points. In contrast, FI produced a more uniform but generally drier soil moisture profile at V6, with relatively flat spatial gradients and lower absolute water content across most sampling positions.

The hydrological contrast between treatments evolved dynamically across years and growth stages. Under DI, the high-moisture zone expanded vertically and horizontally from 2019 to 2021, reflecting cumulative water application effects and reduced evaporative demand in wetter years. At the R3 stage, DI retained higher soil water content in the 20–40 cm depth interval across all three years, whereas FI showed progressive moisture depletion in upper layers, particularly in 2019 and 2020. By 2021, the spatial divergence between treatments attenuated, with both regimes converging toward similar moisture patterns in the deep soil profile (>30 cm). These contrasting moisture architectures—localized high-intensity wetting under DI versus broad low-intensity distribution under FI—establish the hydrological boundary conditions that differentially structured root architectural plasticity (Section 3.2).

### 3.2. Drip Irrigation Induces a Shallow-Dense Root Phenotype with Steeper Vertical Gradients

**Figure 4:**
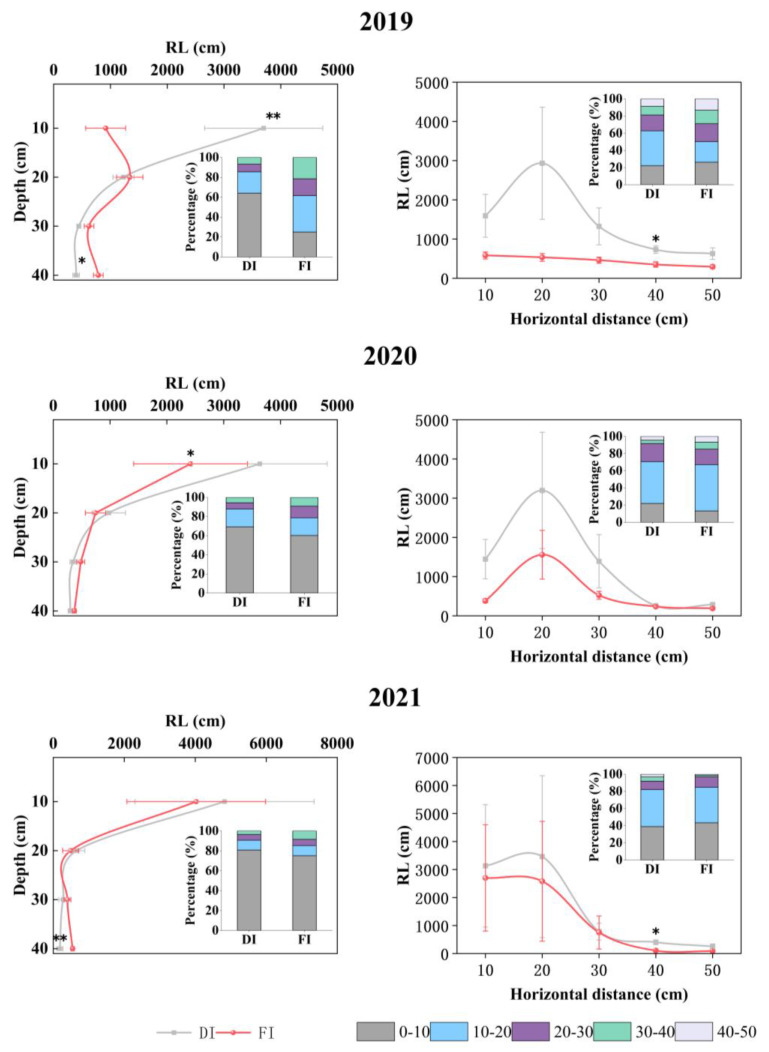
Spatial distribution of maize root length density at the V6 stage as influenced by soil depth and horizontal distance from the plant row, 2019–2021. Asterisks indicate significant differences at * *p* < 0.05 and ** *p* < 0.01.

Comprehensive spatial characterization of RLD at the V6 phenological stage demonstrated distinct root architectural plasticity in response to variable irrigation regimes over the three hydrological years (Figure 4; percentage differences in spatial RLD are calculated relative to FI values for each year, with overall density parameters reported in Table 2). Vertically, RLD under both treatments followed an exponential decay pattern with increasing soil depth. However, DI exhibited a significantly steeper gradient: RLD at 10 cm depth was 303.4% (2019) and 50.4% (2020) higher under DI than under FI, respectively (*p* < 0.05), whereas FI maintained an advantage in deep soil layers, with RLD at 40 cm depth being 49.1% (2019) and 66.1% (2021) higher than DI in 2019 and 2021, respectively (*p* < 0.05). Horizontally, RLD under DI displayed a unimodal distribution, peaking at 20 cm from the drip tape, and maintained relatively high values across all horizontal distances. This was particularly significant at 40 cm horizontal distance in 2019 and 2021, where RLD was 108.4% (2019) and 286.5% (2021) higher under DI than under FI, respectively (*p* < 0.05). In terms of proportional distribution, DI concentrated 78.2% of total fine and medium root length in the vicinity of emitters (0–20 cm horizontal distance), while FI showed a more uniform root distribution and achieved a higher relative proportion in the distant zone (30–50 cm from the planting row). These distinct patterns indicate that DI optimizes rhizosphere resource capture via intensive root proliferation in shallow soil, whereas FI enhances exploration capacity through a deeper and more dispersed root distribution.

### 3.3. Root Density Parameters

DI maximized root establishment during early growth periods, particularly under water deficit conditions, though its relative advantage progressively attenuated with increasing water availability and even reversed at later phenological stages under sufficient moisture. At the V6 stage, drip irrigation-mediated enhancements in RLD, RSAD, and root volume density (RVD) declined precipitously across the hydrological gradient: in the dry year (2019), DI elevated RLD, RSAD, and RVD by 224.6%, 205.1%, and 228.2%, respectively (*p* < 0.01); in the moderate-water year (2020), respective increments decreased to 127.3%, 143.0%, and 117.2% (*p* < 0.01); in the wet year (2021), only RLD remained significantly elevated (+29.1%, *p* < 0.05), with RSAD and RVD exhibiting no significant divergence from control. Notably, at the R3 stage in 2021, FI superseded DI in RLD promotion (+117.2%, *p* < 0.05), suggesting that photosynthate reallocation to reproductive organs under ample water supply may diminish under drip irrigation early-stage architectural advantages. These findings necessitate adaptive management protocols to sustain DI benefits across variable hydrological scenarios.

### 3.4. Spatiotemporal Distribution of Root Density

**Figure 5:**
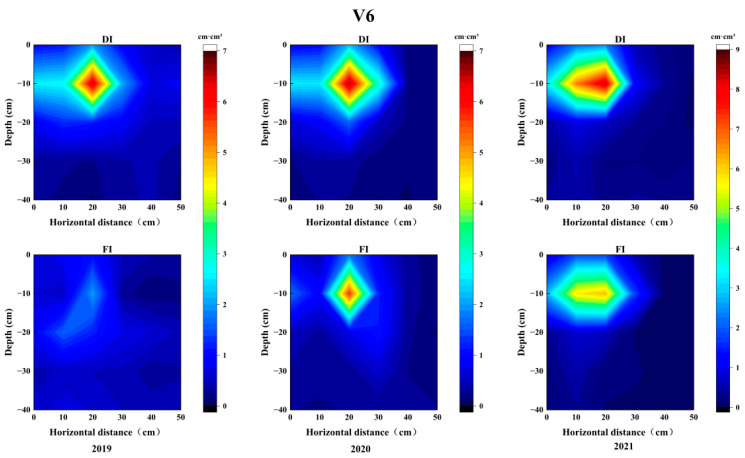

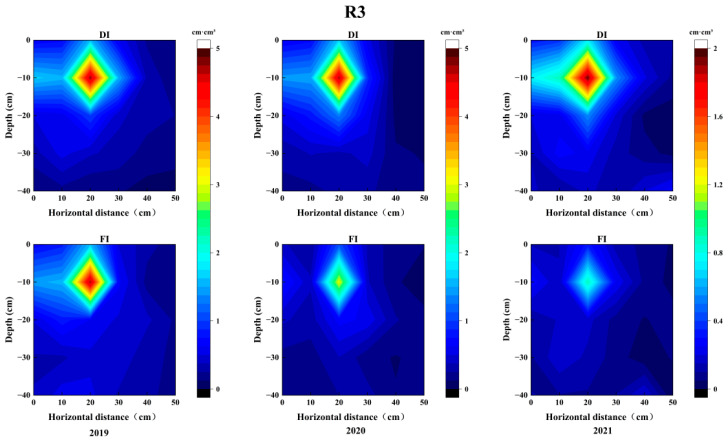
Comparison of the spatial distribution of root density at V6 and R3 stages of seed-maize under DI and FI. V6 stands for the six-leaf stage and jointing stage, while R3 represents the milk stage.

Spatial distribution characteristics of maize root density showed significant irrigation-dependent differences across three hydrological years. Under DI, root density exhibited a typical unimodal distribution in the horizontal direction, peaking at 15–20 cm from the plant base, and was vertically concentrated in the 0–20 cm soil layer at the V6 growth stage (Figure 5). This pattern was particularly prominent in 2020, where root density in this concentrated region under DI was markedly higher than that under FI. In contrast, the FI system promoted greater root biomass allocation to deeper soil layers (20–40 cm) and the horizontal distant zone (>30 cm from the plant base) during 2019–2020. By the end of the study (2021), DI root density expanded horizontally to the 0–30 cm range while maintaining vertical concentration in the 0–20 cm layer, forming a characteristic elliptical distribution centered on the maize stalk. Conversely, FI roots presented a heterogeneous spatial pattern, with significantly lower RLD values at all sampling positions. These results demonstrate that irrigation methods exert distinct shaping effects on root system architecture: DI facilitates concentrated root growth in shallow to middle soil layers, whereas FI promotes deeper and more dispersed exploration of the root zone. Collectively, the three-year data reveal that the spatial divergence between DI and FI root systems is most pronounced at the V6 stage under drought conditions, attenuating under ample moisture and at later reproductive stages (Table 2; Figure 5).

### 3.5. Root Morphological Traits and Root Length Diameter Distribution

**Figure 6:**
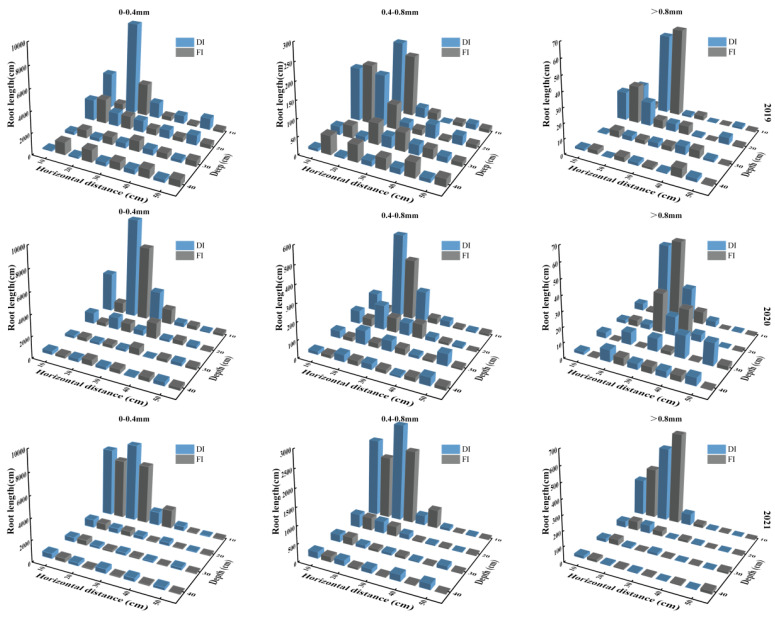
Spatiotemporal distribution of maize root systems categorized by diameter (Coarse roots: >0.8 mm; Medium roots: 0.4–0.8 mm; Fine roots: 0–0.4 mm).

Three-dimensional root diameter class distribution characteristics revealed distinct plasticity differences in root system architecture under various irrigation regimes (Figure 6). Under DI, fine roots (0–0.4 mm) and medium roots (0.4–0.8 mm) exhibited obvious aggregation in the shallow vertical layer (0–10 cm depth) and the horizontal zone proximal to emitters (0–20 cm from the drip tape). Their maximum root lengths in 2020 reached 9784.3 cm and 498.6 cm, respectively, representing increases of 35.0% and 38.6% compared with FI. This “fine and dense” root phenotype concentrated 78.2% of total fine and medium root length within the 0–20 cm soil layer. In contrast, FI promoted a more favorable spatial distribution of coarse roots (>0.8 mm), especially in deep soil (>20 cm) and the distant zone (>30 cm from the plant base). Both treatments displayed a typical exponential decay pattern and L-shaped vertical distribution. However, DI showed a significantly steeper decay gradient (root length at 10 cm depth was 303.4% and 50.4% higher in DI than in FI during 2019 and 2020, respectively, *p* < 0.05), whereas FI maintained a relative advantage in deep soil (root length at 40 cm depth was 49.1% and 66.1% higher in FI than in DI during 2019 and 2021, respectively). Horizontally, both treatments showed a unimodal distribution with a peak at 20 cm. DI consistently maintained greater root length in the near zone (at 40 cm horizontal distance, root length was 108.4% and 286.5% higher in DI than in FI during 2019 and 2021, respectively, *p* < 0.05) but declined more rapidly in the far zone. FI sustained better root distribution in the marginal zone (30–50 cm from the plant base).

### 3.6. Root Restructuring Is Associated with Irrigation-to-Yield Variation

**Figure 7:**
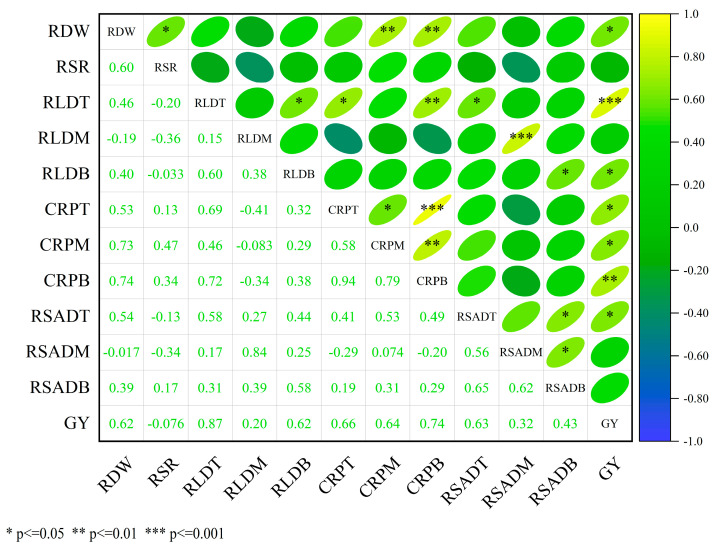
Relationships between maize root traits and grain yield. Note: RDW, root dry weight; R/S, root-to-shoot ratio; RLDT, RLDM, RLDB, root length density in the top, middle, and bottom soil layers, respectively; CRPT, CRPM, CRPB, coarse root proportion in the top, middle, and bottom soil layers, respectively; RSADT, RSADM, RSADB, root surface area density in the top, middle, and bottom soil layers, respectively. For correlation analysis, the four sampling layers were grouped into three composite layers: top (0–20 cm), middle (20–30 cm), and bottom (30–40 cm). Each ellipse represents the correlation between two variables. The orientation of the ellipse indicates the direction of the relationship (right-up for positive, left-up for negative), and color intensity reflects the strength of the correlation.

Correlation analysis revealed that maize yield was most strongly associated with topsoil root length density (RLDT, 0–20 cm, R^2^ = 0.87, *p* < 0.001), followed by bottom-layer coarse root proportion (RLDB, 30–40 cm, R^2^ = 0.74, *p* < 0.01). However, this correlative evidence does not establish root restructuring as the exclusive mechanistic pathway to yield. The continuous water supply maintained by drip irrigation—particularly during reproductive growth—may represent an independent or interacting driver, with root architecture and water delivery continuity operating as co-responding rather than hierarchically ordered factors (Figure 7). This interpretation is supported by the 2021 R3 stage reversal, where FI achieved 117.2% higher RLD than DI (*p* < 0.05, Table 2) yet maintained comparable yield, suggesting that sustained water availability during grain filling may compensate for or override early-stage architectural advantages. Thus, spatial root–water congruence should be regarded as a robust integrative indicator of irrigation productivity rather than proof of a sole causal mechanism.

### 3.7. Root-Water Spatial Coupling

The Sørensen similarity index (SSI) between RLD and SWC distributions was significantly higher under mulched DI than under FI at both growth stages (Table 3). Averaged across years, DI achieved SSI = 0.72 ± 0.06, indicating strong spatial congruence between the concentrated root system and the localized wetting front created by drip emitters, whereas FI showed lower SSI = 0.51 ± 0.09 (*p* < 0.01), reflecting the spatial mismatch between the dispersed water distribution from furrow flooding and the relatively uniform root deployment. By the R3 stage, DI SSI declined to 0.58 ± 0.08 (*p* < 0.05 vs. V6) as the wetting front expanded beyond the emitter-proximal root zone, while FI remained stable at 0.49 ± 0.07 (*p* > 0.05). The consistently higher SSI under DI at V6 (mean ΔSSI = +0.20 across years) demonstrates that localized water delivery from DI enhances spatiotemporal root-water coupling, which mediates the irrigation-to-yield pathway (Section 3.6). The attenuation of this advantage by R3 suggests that the coupling effect is phenologically dependent.

## 4. Discussion

### 4.1. Irrigation Regime Drives Spatial Redistribution of Root Length Density

DI induces a shallow-concentrated, fine-root-dominated architectural phenotype that maximizes resource capture within localized wetted volumes, whereas FI promotes a deeper, spatially dispersed root system optimized for extensive soil exploration. These divergent architectural trajectories reflect distinct morphological plasticity responses to contrasting hydrological boundary conditions (Figure 3). Quantitative analysis revealed DI-mediated proliferation of fine-diameter roots (0–0.4 mm) and medium-diameter roots (0.4–0.8 mm), with respective increments of 35.0% and 38.6% in the 0–10 cm layer adjacent to emitters, concentrating 78.2% of absorptive root length within the 0–20 cm depth interval. This spatial aggregation constitutes an adaptive morphological response to localized water application, consistent with documented high plasticity of maize root systems under heterogeneous environmental conditions [11,12,18,29]. The predominance of fine-root biomass (averaging 85.9% of total root length) aligns with Fiorini et al. [32], who established that root diameter classes govern crop water and nutrient acquisition capacity, and with Eissenstat [33], who demonstrated the physiological advantages of fine-root proliferation.

Both irrigation regimes exhibited characteristic exponential decline in root length density with depth, conforming to the “L-shaped” vertical distribution model [34,35]. However, DI manifested significantly steeper decline gradients, with 303.4% and 50.4% greater root length than FI at 10 cm depth in 2019 and 2020, respectively (*p* < 0.05). This vertical compression maximizes root density within the wetted soil volume under drip emitters, facilitating intensive resource capture in the 0–20 cm irrigation hotspot [6,14]. Horizontally, DI displayed unimodal distributions peaking at 20 cm from the drip line, with consistently elevated RLD across all distances-most prominently 108.4% (2019) and 286.5% (2021) greater than FI at 40 cm horizontal distance (*p* < 0.05). Conversely, FI maintained higher RLD at the 40 cm sampling boundary reflect broader, more dispersed wetting patterns that promote exploratory root growth, as documented by Qin et al. [23] for maize under differential tillage systems. This spatial aggregation likely reflects active hydrotropism toward localized high water potential zones and hydropatterning of lateral roots toward the emitter-proximal wetting front, rather than passive growth in available water.

### 4.2. Root Density Parameters and Spatiotemporal Dynamics

Root density parameters (RLD, RSAD, RVD) exhibited distinct irrigation × growth stage × year interactions (Table 2; Figure 5). At V6, DI consistently enhanced root density with effect magnitudes scaling inversely with water availability: under drought (2019), RLD, RSAD, and RVD increased by 224.6%, 205.1%, and 228.2% (*p* < 0.01); under moderate moisture (2020), gains attenuated to 127.3%, 143.0%, and 117.2% (*p* < 0.01); under ample moisture (2021), only RLD showed significant increase (+29.1%, *p* < 0.05). This context-dependent efficacy indicates that localized water supply alleviates root growth constraints particularly under water deficit, supporting that enhancing soil water-root growth coordination improves maize yield [4,29].

Spatially, DI concentrated roots in the near-irrigation zone (0–20 cm horizontal, 0–20 cm vertical) at V6, exceeding FI by 35–40%. By R3, DI maintained higher overall density while expanding vertically into 20–30 cm depths, forming an elliptical distribution. Critically, at R3 in 2021’s water-sufficient conditions, FI significantly outperformed DI (RLD +117.2%, *p* < 0.05). This reversal suggests that the metabolically intensive fine-root network under DI becomes carbon-limited during reproductive growth when assimilates divert to grain filling [1], whereas under FI, deeper, coarser roots offer maintenance advantages. This temporal decoupling underscores the necessity of stage-specific irrigation management [8,27]. This reversal likely reflects dual mechanisms: (1) carbon limitation of metabolically intensive fine roots under DI as assimilates divert to grain filling, and (2) the compensatory effect of sustained water supply during reproductive growth.

### 4.3. Root Morphological Traits and Diameter-Class Distribution

Contrasting irrigation regimes induce divergent three-dimensional root architectural phenotypes with distinct ecophysiological trade-offs: DI optimizes the root-soil interaction interface through shallow-concentrated, fine-root proliferation to maximize resource acquisition, whereas FI prioritizes mechanical stability via deeper, dispersed coarse-root development to enhance anchorage and lodging resistance. Three-dimensional architectural analysis revealed marked plasticity responses under differential water management (Figure 6). Under DI, fine-diameter (0–0.4 mm) and medium-diameter (0.4–0.8 mm) roots exhibited pronounced spatial aggregation in shallow soil layers proximal to emitters, achieving maximum lengths of 9784.3 cm and 498.6 cm, respectively, in 2020. This phenotype maximizes the root-soil interaction interface for water and nutrient acquisition [9,10]. Conversely, FI promoted superior coarse-root (>0.8 mm) development, particularly in deeper soil profiles (>20 cm) and distal horizontal zones (>30 cm), thereby enhancing mechanical anchorage and lodging resistance.

This architectural differentiation carries clear functional implications: coarse roots provide structural stability while fine roots govern uptake potential [10,19]. Although DI reduced coarse-root proportional biomass, the marked accumulation of fine- and medium-diameter roots substantially expanded total absorptive surface area, corroborating that mulched drip irrigation enhances plant nutritional status through architectural optimization [17,25].

### 4.4. Root Restructuring Is Associated with Irrigation-Induced Yield Variation

Root spatial distribution, rather than total biomass accumulation, was the strongest predictor of water productivity under precision irrigation systems. This pattern is consistent with the hypothesis that shallow, dense fine-root proliferation contributes to yield by enhancing resource capture within the wetted volume. However, because our evidence is correlational, we cannot exclude the possibility that the continuous water supply provided by drip irrigation—particularly during the reproductive stage—represents an independent or interacting driver of yield. Under this alternative interpretation, root restructuring reflects an adaptive plastic response that is spatially congruent with water availability, rather than the sole mechanistic pathway from irrigation to yield.

Spatial distribution analysis revealed that topsoil (0–20 cm) RLD emerged as the dominant predictor of yield formation (R^2^ = 0.87, *p* < 0.001), substantially outperforming bottom-layer coarse-root proportion (30–40 cm) (R^2^ = 0.74), upper- and middle-layer coarse-root proportion (R^2^ = 0.66–0.64), and root surface area density (R^2^ = 0.63). These relationships establish shallow, dense rooting—rather than maximal biomass investment—as the critical determinant of water productivity, suggesting a potential shift in management perspective from uniform water delivery to precision irrigation that indirectly shapes root architecture [1,5].

Fine-root predominance (85.9% of total length) drives this relationship, as these high-specific-surface roots maximize the soil exploration interface for resource acquisition [10,32]. DI-induced fine-root proliferation enhances uptake capacity despite reduced coarse-root allocation, while deep coarse roots provide drought-resistance complementarity (R^2^ = 0.74) via access to deep soil water reserves [30]. The negative yield correlation with root-to-shoot ratio further indicates efficient carbon allocation decoupled from proportional root biomass increase [1].

These findings highlight that DI indirectly modifies root architecture through localized water distribution, in addition to its primary water-saving function. The 78% root concentration in the 0–20 cm layer enhances early-stage resource capture but carries inherent risks of rhizosphere carbon depletion and salt accumulation. Future system optimization should integrate intelligent monitoring to dynamically adapt emitter placement depth (20–30 cm) and irrigation scheduling intensity (high at V6, moderate at R3) to phenological stage, advancing from water-saving agriculture to crop production with improved root architecture [18,36].

The SSI results mechanistically resolve how the shift from FI to mulched DI enhances yield. Under FI, broad surface flooding creates a dispersed wetting pattern that poorly overlaps with the root distribution (SSI = 0.51 at V6), forcing roots to expend carbon on exploratory growth into dry soil zones. In contrast, DI delivers water directly to the root-dense layer via emitters, creating a localized wetting front that coincides with the zone of maximum root proliferation (SSI = 0.72 at V6). This spatial congruence—quantified as a +0.20 mean ΔSSI advantage for DI—reduces the carbon cost of soil exploration and maximizes water uptake per unit root length, thereby mediating the irrigation-to-yield pathway. The decline in DI SSI from V6 to R3 (0.72 → 0.58) reflects the progressive dissipation of the localized wetting front as cumulative evapotranspiration depletes soil moisture, while FI SSI remains stable (0.51 → 0.49) due to its inherently dispersed water distribution. This temporal decoupling under DI explains the 2021 R3 reversal (Section 3.3) and underscores the need for stage-specific drip management to sustain coupling into reproductive growth. The higher SSI under DI reflects active biological coordination between root deployment and water distribution, reducing futile carbon expenditure on soil exploration rather than merely indicating spatial overlap.

## 5. Conclusions

This study demonstrates that irrigation method transition—from furrow to mulched drip—drives adaptive root architectural restructuring, which mediates yield improvement through enhanced root-water spatial coupling:

(1) Irrigation transition induces systematic architectural switching. DI generates a “shallow-dense” phenotype (35.0–38.6% fine-root increase, 78.2% concentration in 0–20 cm) following exponential vertical decay with steeper gradients than FI, driven by hydrotropic aggregation toward localized wetting fronts.
(2) Irrigation effects are hydrologically conditional but directionally consistent. DI advantages scale from +224.6% (drought) to +29.1% (ample moisture) RLD increase, with potential R3 reversal, indicating that water availability regulates plasticity magnitude, but irrigation method sets architectural strategy.
(3) Root restructuring is strongly associated with yield formation, but the available correlative evidence does not establish it as the exclusive mechanistic pathway. Topsoil RLD accounted for the largest share of yield variance (R^2^ = 0.87), indicating that spatial root-water congruence is a robust integrative indicator of irrigation productivity. Nevertheless, the continuous water supply maintained by drip irrigation-especially during reproductive growth-may represent an independent or interacting driver that operates alongside root architectural adjustments. Deep coarse roots appear to provide complementary hydraulic safety under both irrigation regimes.
(4) Irrigation management may benefit from considering root architectural responses as a secondary outcome of precision water application [37].

## Figures and Tables

**Table 1:** Soil physicochemical properties in the experimental site.

Soil Depth (cm)	SOM (g/kg)	NO_3_^−^-N (mg/kg)	NH_4_^+^-N (mg/kg)	AP (mg/kg)	AK (mg/kg)	pH	EC (ds/cm)	TSC (g/kg)
0–10	16.79	15.27	10.62	26.52	415.98	8.19	0.23	0.65
10–20	17.92	16.88	11.32	26.76	416.78	8.20	0.29	0.35
20–30	16.74	14.22	9.82	23.56	354.65	8.16	0.30	0.55
30–40	8.16	11.40	7.20	8.13	246.37	8.14	0.38	0.95

Note: Soil Organic Matter (SOM), Nitrate Nitrogen (NO_3_^−^-N), Ammonium Nitrogen (NH_4_^+^-N), Available Phosphorus (AP), Available Potassium (AK), Electrical Conductivity (EC), Total Salt Content (TSC). Soil organic matter (SOM) was determined by potassium dichromate oxidation; NO_3_^−^-N and NH_4_^+^-N by KCl extraction and continuous flow analyzer; available P and K by NaHCO_3_ and NH_4_OAc extraction, respectively; pH and EC in 1:5 soil-water suspension; total salt content by residue drying method.

**Table 2:** Comparison of root density between DI and FI treatments at the V6 and R3 stages from 2019 to 2021.

Year	Period	Treatment	RLD (cm/cm^3^)	RSAD (cm^2^/cm^3^)	RVD (cm^3^/m^3^)
2019	V6	DI	0.994 ± 0.230 **	0.113 ± 0.025 **	1.187 ± 0.292 **
		FI	0.306 ± 0.026	0.037 ± 0.003	0.362 ± 0.034
	R3	DI	0.656 ± 0.167	0.075 ± 0.020	0.652 ± 0.225
		FI	0.632 ± 0.078	0.075 ± 0.011	0.762 ± 0.146
2020	V6	DI	0.907 ± 0.253 **	0.145 ± 0.043 **	2.161 ± 0.766 **
		FI	0.399 ± 0.100	0.060 ± 0.018	0.797 ± 0.303
	R3	DI	0.619 ± 0.166	0.079 ± 0.023	0.855 ± 0.270
		FI	0.693 ± 0.192	0.096 ± 0.031	1.221 ± 0.475
2021	V6	DI	1.109 ± 0.497 *	0.148 ± 0.069	1.748 ± 0.910
		FI	0.859 ± 0.403	0.109 ± 0.050	1.137 ± 0.512
	R3	DI	0.145 ± 0.041	0.026 ± 0.008	0.411 ± 0.131
		FI	0.315 ± 0.009 *	0.047 ± 0.019	0.613 ± 0.286

Note: Values within a column followed by an asterisk and two asterisks are significantly different at *p* < 0.05 and *p* < 0.01. V6 stands for the six-leaf stage and jointing stage, while R3 represents the milk stage.

**Table 3:** Sørensen similarity index (SSI) between root length density and soil water content distributions under mulched DI and FI. Means ± SE, n = 3. ** *p* < 0.01, * *p* < 0.05.

Year	Stage	DI	FI
2019	V6	0.74 ± 0.05 **	0.48 ± 0.08
2019	R3	0.61 ± 0.07 *	0.47 ± 0.09
2020	V6	0.73 ± 0.06 **	0.53 ± 0.07
2020	R3	0.59 ± 0.09	0.51 ± 0.08
2021	V6	0.68 ± 0.08 *	0.55 ± 0.10
2021	R3	0.52 ± 0.11	0.50 ± 0.09

## Data Availability

The datasets obtained during this study are accessible from the corresponding author upon reasonable request.

## References

[1] Harrison M.T., Tardieu F., Dong Z., Messina C.D., Hammer G.L. (2014). Characterizing drought stress and trait influence on maize yield under current and future conditions. Glob. Change Biol..

[2] IPCC (2022). Climate Change 2022: Impacts, Adaptation and Vulnerability.

[3] Wang J. (2023). Distribution and evolution characteristics of drought under the background of warming and wetting climate in Xinjiang. Arid Environ. Monit..

[4] Gao J., Zhang Y., Xu C., Wang P., Huang S., Lv Y. (2024). Enhancing spatial and temporal coordination of soil water and root growth to improve maize (*Zea mays* L.) yield. Agric. Water Manag..

[5] Lobell D.B., Roberts M.J., Schlenker W., Braun N., Little B.B., Rejesus R.M., Hammer G.L. (2014). Greater sensitivity to drought accompanies maize yield increase in the U.S. Midwest. Science.

[6] Cao Y., Cai H., Sun S., Gu X., Mu Q., Duan W., Zhao Z. (2022). Effects of drip irrigation methods on yield and water productivity of maize in Northwest China. Agric. Water Manag..

[7] Song Q., Zhang F., Li X., Yue S., Luo Z., Li S. (2024). Understanding of maize root responses to changes in water status induced by plastic film mulching cultivation on the Loess Plateau, China. Agric. Water Manag..

[8] Han Y., Qiao D., Lu H. (2023). Spatial-temporal coupling pattern between irrigation demand and soil moisture dynamics throughout wheat-maize rotation system in the North China Plain. Eur. J. Agron..

[9] Hodge A., Berta G., Doussan C., Merchan F., Crespi M. (2009). Plant root growth, architecture and function. Plant Soil.

[10] Lynch J.P. (2011). Root phenes for enhanced soil exploration and phosphorus acquisition: Tools for future crops. Plant Physiol..

[11] Yu P., Hochholdinger F., Li C. (2019). Plasticity of lateral root branching in maize. Front. Plant Sci..

[12] Liu Y.X., Sun J.-H., Zhang F.-F., Li L. (2020). The plasticity of root distribution and nitrogen uptake contributes to recovery of maize growth at late growth stages in wheat/maize intercropping. Plant Soil.

[13] Schneider H.M., Klein S.P., Hanlon M.T., Nord E.A., Kaeppler S., Brown K.M., Warry A., Bhosale R., Lynch J.P. (2020). Genetic control of root architectural plasticity in maize. J. Exp. Bot..

[14] Coelho E.F., Or D. (1999). Root distribution and water uptake patterns of corn under surface and subsurface drip irrigation. Plant Soil.

[15] Dong H., Kong X., Luo Z., Li W., Xin C. (2010). Unequal salt distribution in the root zone increases growth and yield of cotton. Eur. J. Agron..

[16] Chen X., Zhang J., Chen Y., Li Q., Chen F., Yuan L., Mi G. (2014). Changes in root size and distribution in relation to nitrogen accumulation during maize breeding in China. Plant Soil.

[17] Zhang L., Meng Y., Li S., Yue S. (2020). Film mulching optimizes the early root and shoot development of rain-fed spring maize. Agron. J..

[18] Jin M., Liu H., Liu X., Guo T., Guo J., Yin Y., Ji Y., Li Z., Zhang J., Wang X. (2023). Complex genetic architecture underlying the plasticity of maize agronomic traits. Plant Commun..

[19] Li C., Guo J., Wang D., Chen X., Guan H., Li Y., Zhang D., Liu X., He G., Wang T. (2023). Genomic insight into changes of root architecture under drought stress in maize. Plant Cell Environ..

[20] Dietrich D. (2018). Hydrotropism: How roots search for water. J. Exp. Bot..

[21] Orosa-Puente B., Leftley N., von Wangenheim D., Banda J., Srivastava A.K., Hill K., Truskina J., Bhosale R., Morris E., Srivastava M. (2018). Root branching toward water involves posttranslational modification of transcription factor ARF7. Science.

[22] Shkolnik D., Nuriel R., Bonza M.C., Costa A., Fromm H. (2018). MIZ1 regulation of ECA1 generates a slow, long-distance Ca^2+^ signal necessary for root hydrotropism. Proc. Natl. Acad. Sci. USA.

[23] Qin R., Noulas C., Herrera J.M. (2018). Morphology and distribution of wheat and maize roots as affected by tillage systems and soil physical parameters in temperate climates: An overview. Arch. Agron. Soil Sci..

[24] Jin X., Chen M., Fan Y., Yan L., Wang F. (2018). Effects of mulched drip irrigation on soil moisture and groundwater recharge in the Liao River Plain, China. Water.

[25] Wang J., Du G., Tian J., Jiang C., Zhang Y., Zhang W. (2021). Mulched drip irrigation increases cotton yield and water use efficiency via improving fine root plasticity. Agric. Water Manag..

[26] Qu Z., Chen Q., Yin S., Feng H., Liu Y., Li C. (2024). Effects of drip irrigation coupled with controlled release potassium fertilizer on maize growth and soil properties. Agric. Water Manag..

[27] Ma Y., Ren J., Yang S., Ding R., Du T., Kang S., Tong L. (2025). Enhancing maize yield and water productivity through coordinated root-shoot growth under mild water stress in dense planting. Field Crops Res..

[28] Liu M., Liang F., Li Q., Wang G., Tian Y., Jia H. (2023). Enhancement growth, water use efficiency and economic benefit for maize by drip irrigation in Northwest China. Sci. Rep..

[29] Singh M., Singh S., Deb S., Ritchie G. (2023). Root distribution, soil water depletion, and water productivity of sweet corn under deficit irrigation and biochar application. Agric. Water Manag..

[30] Dunbabin V.M., Postma J.A., Schnepf A., Pagès L., Javaux M., Wu L., Leitner D., Chen Y.L., Rengel Z., Diggle A.J. (2013). Modelling root-soil interactions using three-dimensional models of root growth, architecture and function. Plant Soil.

[31] MOA (Ministry of Agriculture of China) (2006). Technical Regulations for Maize Yield Estimation (NY/T 1209-2006).

[32] Fiorini A., Boselli R., Amaducci S., Tabaglio V. (2018). Effects of no-till on root architecture and root-soil interactions in a three-year crop rotation. Eur. J. Agron..

[33] Eissenstat D.M. (1992). Costs and benefits of constructing roots of small diameter. J. Plant Nutr..

[34] Gale M.R., Grigal D.F. (1987). Vertical root distributions of northern tree species in relation to successional status. Can. J. For. Res..

[35] Jackson R.B., Canadell J., Ehleringer J.R., Mooney H.A., Sala O.E., Schulze E.D. (1996). A global analysis of root distributions for terrestrial biomes. Oecologia.

[36] Li H.R., Mei X.R., Wang J.D., Hang F., Hao W.P., Li B.G. (2021). Surface drip fertigation significantly increased crop yield, water productivity and nitrogen use efficiency with respect to traditional fertilization practices: A meta-analysis in China. Agric. Water Manag..

[37] Hu T., Kang S., Li F., Zhang J. (2011). Effects of partial root-zone irrigation on hydraulic conductivity in the soil–root system of maize plants. J. Exp. Bot..

